# Near-Instantaneously Self-Healing Coating toward Stable and Durable Electromagnetic Interference Shielding

**DOI:** 10.1007/s40820-021-00709-0

**Published:** 2021-09-08

**Authors:** Lihua Zou, Chuntao Lan, Songlin Zhang, Xianhong Zheng, Zhenzhen Xu, Changlong Li, Li Yang, Fangtao Ruan, Swee Ching Tan

**Affiliations:** 1 Anhui Province International Cooperation Research Center of Textile Structure Composite Materials, Anhui Polytechnic University, Anhui, 241000 Wuhu People’s Republic of China; 2 Department of Materials Science and Engineering, National University of Singapore, 9 Engineering Drive 1, Singapore, 117575 Singapore; 3 Key Laboratory of Eco-Textiles, Ministry of Education, Jiangnan University, Wuxi, 214122 Jiangsu People’s Republic of China; 4 Department of Mechanical Engineering, University of Delaware, Newark, DE 19716 USA

**Keywords:** Electromagnetic interference shielding, Superhydrophobic coating, Multifunctional textiles, Self-healing, Conductive polymer

## Abstract

Durable electromagnetic interference (EMI) shielding is highly desired, as electromagnetic pollution is a great concern for electronics’ stable performance and human health. Although a superhydrophobic surface can extend the service lifespan of EMI shielding materials, degradation of its protection capability and insufficient self-healing are troublesome issues due to unavoidable physical/chemical damages under long-term application conditions. Here, we report, for the first time, an instantaneously self-healing approach via microwave heating to achieve durable shielding performance. First, a hydrophobic 1*H*,1*H*,2*H*,2*H*-perfluorooctyltriethoxysilane (POTS) layer was coated on a polypyrrole (PPy)-modified fabric (PPy@POTS), enabling protection against the invasion of water, salt solution, and corrosive acidic and basic solutions. Moreover, after being damaged, the POTS layer can, for the first time, be instantaneously self-healed via microwave heating for a very short time, i.e*.*, 4 s, benefiting from the intense thermal energy generated by PPy under electromagnetic wave radiation. This self-healing ability is also repeatable even after intentionally severe plasma etching, which highlights the great potential to achieve robust and durable EMI shielding applications. Significantly, this approach can be extended to other EMI shielding materials where heat is a triggering stimulus for healing thin protection layers. We envision that this work could provide insights into fabricating EMI shielding materials with durable performance for portable and wearable devices, as well as for human health care.

**Supplementary Information:**

The online version contains supplementary material available at 10.1007/s40820-021-00709-0.

## Highlights

- Stable and durable electromagnetic interference (EMI) shielding performance was achieved in harsh environments by applying a thin protective coating of hydrophobic 1*H*,1*H*,2*H*,2*H*-perfluorooctyltriethoxysilane (POTS) on polypyrrole (PPy)-modified fabrics.
- The damaged POTS layer can be instantaneously self-healed (~ 4 s) for the first time by the microwave heating effect of PPy.
- The self-healing process is repeatable even after severe damages, highlighting the great potential to exploit EMI shielding materials’ instant microwave heating effects to enable robust and durable EMI shielding applications.

**Supplementary Information:**

The online version contains supplementary material available at 10.1007/s40820-021-00709-0.

## Introduction

The ever-increasing number of electronics and wearable gadgets [1, 2] brings much convenience with the advancement of telecommunication technologies [3–5]. However, concerns about electromagnetic interference (EMI) issues also arise, such as data loss and misinterpretation because of electromagnetic induction effects [6–9]. The potential adverse effects of electromagnetic radiation on human health cannot be ignored [10], especially with the advent of the rapid commercialization of emerging 5G wireless devices that will be deployed everywhere around us [11–14].Consequently, high-performance and durable EMI shielding materials are in high demand and have placed additional requirements on fabrication (e.g., easy processability), performance (e.g., stability and durability for devices working in harsh environments), wearability (e.g., high air permeability and lightweight), etc. Consequently, a wide range of building-block materials, including nanometal products (nanoparticles or nanowires) [15–17], nanocarbon products (carbon nanotubes [18, 19] or graphene [9]), MXene [20], conductive polymers [21, 22], ferrite [23], and their hybrids [24–26], have been investigated for use in the form of a coating on a supporting substrate (e.g., a fabric) for EMI shielding. However, due to unavoidable physical/chemical interruptions under long-term application conditions, achieving durable performance for most EMI shielding materials is still challenging.

The EMI shielding performance of some conductive materials is susceptible to degradation following exposure to salt or corrosive solutions or even water rinsing due to structural disintegration, oxidation, or corrosive reactions [8]. Therefore, a protective layer of organic or inorganic materials, such as polyvinylidene fluoride [27], polyurethane [9, 16], polydimethylsiloxane [23, 28], and silicon [7], has been reported to extend the stability and durability of shielding performance. Among the protection layer features, the hydrophobicity feature has attracted extensive research interest because it directly prevents the intrusion of aqueous solutions into EMI shielding materials, resulting in stable performance in harsh environments [7, 23]. Superhydrophobicity is even more profound to achieve extended lifespan for EMI shielding materials [15]. Generally, a thin protection layer is preferred to balance the trade-off between high shielding performance and effective protection. To take textile-based EMI shielding material as an example, the protection layer thickness is an important factor that should be taken into consideration to favorably retain the fabric’s permeability, softness, and other wearability features (i.e., esthetic design). However, abrasion or chemical damage to the protection layer occurs repeatedly during long-term service, unavoidably degrading the protection effectiveness [29]. To address this issue, approaches to efficiently realize the self-healing ability of this thin protection layer are highly desired.

Self-healing can be obtained via the formation of dynamic chemical bonds (such as reversible Diels–Alder reaction, reversible ester linkage, the exchange of sulfur–sulfur links, and imine exchange reactions) and supramolecular interactions (such as the broken reformation of hydrogen bonding, metal–ligand coordination, and hydrophobic association, etc.) [30]. For superhydrophobic materials, the self-healing process is regarded as the hydrophobic association of supramolecular interaction [31, 32]: low-surface-energy material migrates to the damaged surface due to their tendency to have a low surface energy similar to that of air (enthalpy-driven nature). After a period of time (i.e., tens of minutes or hours), the low-surface-energy material will cover the damaged area and form a new coating surface. However, one issue for these approaches to achieving self-healing is the relatively long healing time, ranging from tens of minutes to hours (depending on the kinetic reactions of the chemical bonds or the kinetic constants of the polymer chain mobility) [33]. Applying external stimuli, such as humidity and heating, will increase the mobility of molecules [34] and speed up the migration of molecules, thus healing the damaged surface [35]. Consequently, selecting an appropriate external stimulus could shorten the molecule mobility and migration time, therefore reducing the healing time to a few minutes. For instance, Sun and co-workers shortened the healing time of fluorinated-decyl polyhedral oligomeric silsesquioxane from 10 h to 5 min by increasing the surrounding humidity (from 35 to 100% relative humidity) [35]. Later, they also achieved similar results (5 min) for the same superhydrophobic coating by exploiting the Joule heating effects of copper-deposited cotton fabric [29]. Although current approaches are sufficient for superhydrophobicity restoration, the long healing time (from minutes to even hours) is troublesome for applications where instant property recovery is highly needed. In this context, a superfast, simple yet robust self-healing strategy to instantaneously restore the effectiveness of the protection layer (i.e., reconstructing the superhydrophobic coating) for stable and durable EMI shielding performance is still under investigation.

Here, we report, for the first time, a near-instantaneously self-healing approach via microwave heating to obtain instant recovery of the protection capability of a thin layer of 1*H*,1*H*,2*H*,2*H*-perfluorooctyltriethoxysilane (POTS) to significantly extend the lifespan of polypyrrole (PPy)-coated fabric for durable EMI shielding applications. First, PPy-coated fabric exhibited an average shielding effectiveness (*SE*) of 24.7 dB in the range of 8.2–12.4 GHz (X-band). Then, a thin yet hydrophobic POTS layer was coated on the PPy-modified fabric to provide adequate protection of the shielding property, even in harsh environments (acidic or basic solutions). More importantly, by taking advantage of the microwave heating effect of PPy, near-instantaneous self-healing (within 4 s) of the thin POTS layer was successfully achieved with high healing efficiency just using only a household microwave oven. Furthermore, this healing process was repeatable after severe mechanical abrasion or chemical damage occurred. Even after ten cycles of damaging (plasma etching)/healing treatments, the healing efficiency was still greater than 96%, with an average *SE* of 23.5 dB remaining, demonstrating the excellent protection capability and EMI shielding durability of our coated fabric. Although the superfast self-healing demonstration in this work is just on a piece of fabric using PPy and POTS, the idea of exploiting the microwave absorption-heating effect could potentially be extended to many other combinations of EMI shielding materials and protective coating layers where microwave heating acts as a self-healing stimulus. This work provides insights into the fabrication of stable and durable EMI shielding materials with a protective surface that possesses an instantaneously self-healing feature via microwave heating.

## Experimental Section

### Materials

Pyrrole (Py, 98.0%) and POTS (98.0%) were purchased from Millipore Sigma (USA). Ferric chloride hexahydrate (FeCl_3_·6H_2_O, ≥ 98.0%), hydrochloric acid (HCl, 37%), sodium hydroxide (NaOH, ≥ 98.0%), sodium chloride (NaCl, ≥ 98.0%), ethanol, and sodium dodecylbenzene sulfonate (SDBS) were ordered from Sinopharm Chemical Reagent Co., Ltd. All chemicals were analytical reagent grade and were used as received without any further purification. Plain woven cotton fabric was provided by Shandong Lutai Co., Ltd.

### Preparation of PPy and PPyn@POTS Fabrics

**Fig. 1:**
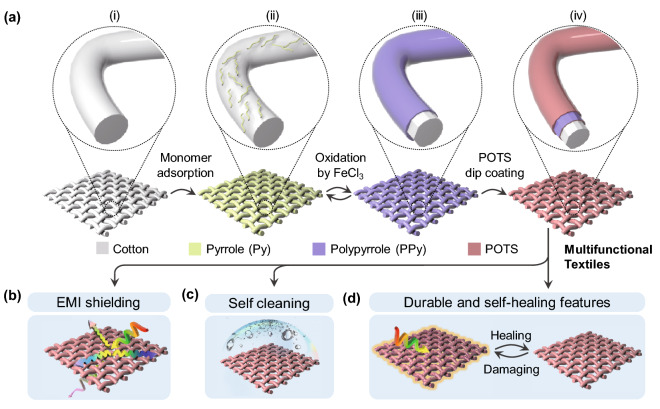
Schematic of the multifunctional PPy*n*@POTS fabric preparation. **a** Fabrication process of PPy*n*@POTS fabrics via a dip-coating approach. The desired number of PPy coating layers can be obtained by repeating the adsorption-oxidation process before the final protection layer coating of POTS. **b–d** The multifunctionality of the coated fabric with high EMI shielding (PPy), self-cleaning (POTS), and durable performance assisted by near-instantaneously self-healing capability

The cotton fabric was first cleaned using ethanol under 15 min of sonication, rinsed with deionized water several times and dried in an oven at 100 ℃. Then, the fabric substrate was dipped into a Py monomer solution (0.6 M in a sodium dodecylbenzene sulfonate solution of 5 mg mL^−1^) for a 5-min adsorption process. Subsequently, the substrate was immersed in a FeCl_3_ aqueous solution (0.8 M in 0.1 M aqueous HCl) for 60 min in an ice bath for the *in situ* polymerization of PPy. Thus, the PPy deposition cycle of adsorption and polymerization can be repeated as needed (denoted as PPy*n*, where *n* = 1, 2, 3…). Afterward, the PPy-modified fabric was washed thoroughly with distilled water and dried in an oven at 80 °C for 3 h. Finally, to obtain a superhydrophobic surface, the PPy-modified fabric was further deposited with a thin layer of POTS via a dip-coating procedure (soaking for 15 min in a POTS solution with a concentration of 40 mg mL^−1^ in ethanol). After drying at 90 ℃ for 15 min, the final sample was obtained and referred to as PPy*n*@POTS (see Fig. 1 for the schematic illustration of the preparation).

### Material Characterizations

The micro- and macro-morphologies of the fabrics were observed using a scanning electron microscope (S4800, Hitachi Japan) and an optical microscope (CX23, Olympus), respectively. The infrared spectra of the fabric, the PPy powder, and PPy-modified fabric were recorded on a Nicolet NEXUS-670 FTIR spectrometer in the 600–4000 cm^−1^ range. The surface chemical compositions of the samples were characterized by X-ray photoelectron spectroscopy (XPS, Escalab 250). Thermogravimetric analysis (TGA) was carried out using a Netzsch TG209F1 at a heating rate of 10 °C min^−1^ from 25 to 600 °C under an air atmosphere. Air permeability tests were conducted using YG461E-III (Wuhan Guoliang Instrument Co., Ltd., China) according to ASTM D737-2018, with a testing sample area of 20 cm^2^ and a pressure difference of 100 Pa. The thickness of the fabrics was tested with a YG141N digital fabric thickness gauge (Natong Hongda Experiment Instruments Co., Ltd., China) according to the standard test method for the thickness of textile materials of ASTM D 1777–96. The flexural rigidity of the fabrics was characterized by a cantilever test using an FY207 bending tester (Wenzhou Fangyuan Instrument Co., Ltd, China) according to ASTM F3260-2017. Surface resistance was evaluated based on four-point probe resistance measurements (RTS-8 model, Four Probe Tech., Guangzhou, China). The water contact angle (CA) was measured using a contact angle meter (OCA15EC, DataPhysics, Germany) with a 3 µL water droplet. The average value of five specimens and deviation were calculated.

### Stability Tests of Electrical Property and EMI Shielding Performance

The surface resistance and *SE* of PPy6@POTS and PPy6 before and after mechanical bending, twisting, and stripping were characterized. The disturbance of various aqueous environments with different chemicals (i.e., DI water, NaCl (1 M), HCl (pH 1), and NaOH (pH 14) solutions) on PPy6@POTS fabrics was assessed by measuring the water CA, surface resistance, and *SE* after different immersion times. The *SE* variation of PPy6@POTS and PPy6 after washing (using household washing machine according to AATCC standard (61–2013 test No. 2A) with OMO liquid detergent (1.5 mg mL^−1^) for 45 min and bath sonication for 2 h with a water temperature of 49 ± 2 °C) was measured to evaluate the washing stability.

### Plasma Damage and Self-healing Using a Microwave

A YZD08 − 5C model of plasma cleaner (Seot (Beijing) Technology Co., Ltd., China) was used to purposely damage the surface of the PPy6@POTS fabric via plasma etching. Plasma treatment is widely used to deliberately damage superhydrophobic surfaces to evaluate their self-healing ability due to its quick and simple process [31, 36]. Then, etched fabrics were put into a microwave oven (Galanz) for irradiation at 2.45 GHz and 100 W. After oven heating, the corresponding surface temperatures and infrared thermal images were recorded using an infrared thermometer (FLIR ThermoVision A40M).

### EMI Shielding Effectiveness Measurement and Calculations

A vector network analyzer (VNA, Agilent N5424A) was used to measure the *SE* in the frequency range of 8.2–12.4 GHz (X-band), which is widely used in many applications, such as Doppler radar, TV signal transmissions, mobile phone relay systems, and other communication technologies [37]. The sample size is fixed at 22.86 × 10.86 mm^2^. *SE*, a parameter to evaluate the shielding performance, is defined as Eq. 1 [38]:

1 $$SE=-10 lg\frac{{P}_{t}}{{P}_{i}}=-10 lg{|{S}_{12}|}^{2}=-10 lg{|{S}_{21}|}^{2}$$

where *S*_12_ and *S*_21_ are the S-parameters recorded from VNA and *P*_t_ and *P*_i_ are the transmitted power and incident power of electromagnetic waves, respectively. Additionally, the reflectivity (*R*) and transmissivity (*T*) of the shield performance were calculated based on Eqs. (2) and (3):

2 $$T = \left| {S_{12} } \right|^{2} = \left| {S_{21} } \right|^{2}$$

3 $$R = \left| {S_{11} } \right|^{2} = \left| {S_{22} } \right|^{2 }$$

The shielding mechanisms of reflection loss (*SE*_*R*_) and absorption loss (*SE*_*A*_) were elaborately calculated according to Eqs. (4) and (5) [16, 38]:

4 $$SE_{R} = - 10lg\left( {1 - R} \right)$$

5 $$SE_{A} = - 10lg[\left( {T/\left( {1 - R} \right)} \right]$$

### COMSOL Simulation of the Surface Temperature of PPy-coated Cotton Yarn

Finite element analysis using COMSOL Multiphysics 5.5 (COMSOL Co., Ltd.) software was conducted to simulate the microwave heating of PPy-coated cotton yarn. To deliver a reasonable result, we first simplified the geometry of the PPy6@POTS fabric based on our experimental observations (Fig. S1) and calculated the microwave heating effect of single cotton yarn with a PPy coating (Fig. 5b). The schematic of the model setup is shown in Fig. S1d. Using the built-in Multiphysics coupling within COMSOL’s software, both electromagnetic wave and heat transfer in solid modules were solved simultaneously using Eqs. (6) and (7):

6 $$\nabla \times {\mu }_{r}^{-1}\left(\nabla \times E\right)-{k}_{0}^{2}\left({\varepsilon }_{r}-\frac{j\sigma }{\omega {\varepsilon }_{0}}\right)E=0$$

7 $$\rho {C}_{p}\frac{\partial T}{\partial t}-\nabla \cdot \left(k\nabla T\right)= Q(t)$$

where *ε*_0_ is the vacuum permittivity, *k*_0_ is the wavenumber in a vacuum, and *ε*_r_, *μ*_r_, and σ are the relative permittivity, the relative permeability, and the electrical conductivity of the PPy coating and cotton yarn, respectively. *T* is the temperature; *ρ*, *C*_p_, and k are the density, heat capacity, and heat transfer coefficient of the heated yarn, respectively. The walls of the microwave oven are assumed to be perfect electrical conductors with the following boundary conditions:

8 $$n\times E=0$$

The boundary conditions in the heat transfer module consisted of thermal insulation at both ends of the coated yarn, convection heat flux on the coated yarn surface, and diffuse surface at the interface between the PPy coating layer and cotton yarn:

9 $$-n\cdot q=0$$

10 $$-n\cdot q={h}_{c}(T-{T}_{air})$$

11 $$-n\cdot q=\varepsilon \sigma \left(T-{T}_{cotton}\right)$$

The built-in frequency-transient study was utilized to solve the equations where the temperature data of coated yarn were recorded at 0.1 s intervals over the range from 0 to 4 s.

## Results and Discussion

### Morphology and Structural Characterization of PPyn@POTS Cotton Textiles

Among various functionalities, the textiles’ EMI shielding performance has drawn significant attention due to its unique importance for personal health care in the proximity of electromagnetic pollution. Cotton fabric, composed of the most used natural fiber, was chosen as the substrate for the coating. PPy, an intrinsically conductive polymer, is a promising EMI shielding material considering its high conductivity, long-term stability, and easy synthesis route [7, 21, 22]. With this in mind, the *in situ* polymerization of PPy on the supporting substrate of cotton fabric via* a* dip-coating process was carried out (as shown in Fig. 1a). The dip-coating method, for multifunctional textile preparation, displays many merits over others (e.g., solution or drop casting [27], screen printing [9], and vacuum filtration [39]), including (1) suitability for various building blocks with different 1-dimensional (1D), 2D, and 3D shapes; (2) controllable thickness via merely adjusting the number of dip-coating cycles or the concentration of the dip-coating solution; and (3) greatly retaining the flexibility and breathability of the original textiles [29]. Py monomer adsorption and oxidation were conducted on fabric through two separate steps (Fig. 1a i–iii) to prevent excessive reactions (i.e., agglomerations of PPy). Then, an additional protective coating of the hydrophobic thin POTS layer (Fig. 1a iv) was deposited on the PPy*n* (n represents the deposition cycles of PPy)-modified fabric via dip coating (hereafter referred to PPy*n*@POTS). POTS is a commonly used hydrophobic agent because of its low surface energy. Additionally, when the coated surface is rough and has a porous structure, applying a POTS coating can easily construct a superhydrophobic surface, which improves the stability and durability of the beneath functional coating during exposure to aqueous, alkaline, and acidic solutions, as mentioned in the Introduction section [40]. Furthermore, via microwave oven heating, the damaged superhydrophobic coating shows a self-healing ability and can be instantaneously repaired within a few seconds, which greatly extends the lifespan of EMI materials in extreme-environment applications. Thus, the combination of POTS and conductive PPy endows the cotton fabric with multiple functions, including EMI shielding, self-cleaning, and durable/self-healing properties (Fig. 1b–d).

**Fig. 2:**
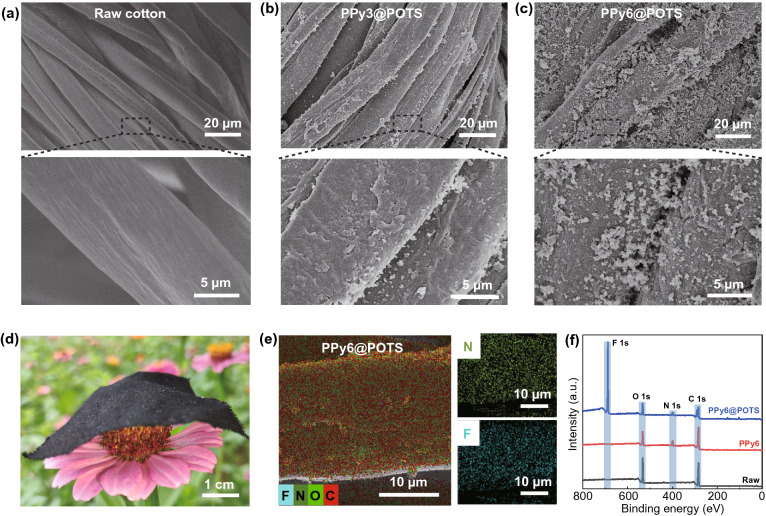
Morphologies and elemental compositions of PPy*n*@POTS fabrics. **a–c** SEM images of **a** raw, **b** PPy3@POTS, and **c** PPy6@POTS fabrics. **d** Photograph of a swatch of PPy6@POTS cotton fabric draped on a flower. **e** EDS elemental mapping of PPy6@POTS fabric, showing the uniform distribution of the PPy and POTS coatings. **f** XPS spectra of raw, PPy6 and PPy6@POTS fabrics

Figure 2a–c shows the surface morphologies of fabrics with and without the PPy*n*@POTS coating. The control (i.e., bare cotton fabric) presented a clean fiber surface with grooves and veins (Fig. 2a). A rough topography on each fiber surface was observed for PPy3@POTS (Fig. 2b). Interestingly, the additional POTS layer did not alter the fiber surface morphology compared to that of the PPy3-coated fabric (Figs. S2a and S3), possibly indicating the thin thickness of POTS as a protective layer (Fig. S3). The successful coating of PPy can be further verified by Fourier transform infrared (FTIR) spectrum data (Fig. S4), where several unique peaks were observed and assigned to C-N stretching (at 1295 cm^−1^), C = N stretching (at 1151 cm^−1^), N–H stretching (at 1088 cm^−1^), and N–H wagging (at 1033 cm^−1^) [22]. By further repeating the deposition cycles of PPy to 6, the fiber surface of PPy6@POTS became even rougher (Fig. 2c) than that of PPy3@POTS. It is worth pointing out that although more deposition cycles of PPy would facilitate enhancing the EMI shielding performance, the functionalities of EMI shielding textiles as building blocks for clothing may be compromised (i.e., decreased flexibility or breathability, increased area mass density, etc.). In our study, the fabric flexural rigidity of PPy*n*@POTS continuously increased as the number of PPy deposition cycles increased, reaching 0.051 cN cm^2^ cm^−1^ for PPy6@POTS (Fig. S5). Nevertheless, this value still indicated good flexibility and was comparable to that of other common textiles, including fabrics in commercial suits (0.080 cN cm^2^ cm^−1^) and shirts (0.050 cN cm^2^ cm^−1^) [38, 41], flame-retardant fabrics (0.092 cN cm^2^ cm^−1^) [35], and superhydrophobic fabrics (0.110 cN cm^2^ cm^−1^) [29]. Additionally, the area mass density of the PPy6@POTS fabric was 12.633 mg cm^−2^ (Fig. S6), which was still lightweight (Fig. 2d) and even lighter than most other multifunctional textiles, such as stretchable and durable EMI shielding fabric (~ 15.660 mg cm^−2^) [16], self-healing and superhydrophobic fabric (~ 15.790 mg cm^−2^) [35], and conductive and superhydrophobic fabric (~ 29.700 mg cm^−2^) [28]. Although the decoration of the functional coatings slightly reduced the air permeability of the base fabric (Fig. S7a), the value for our samples is comparable to those of other EMI shielding fabrics. Specifically, the PPy6@POTS fabric retained a good air permeability of 283.8 mm s^−1^. Lower permeability was reported for some other EMI shielding textiles, such as PPy/MXene fabric (~ 130 mm s^−1^) [7], fluorinated material/Cu-coated fabric (187.8 mm s^−1^) [29], poly(dimethylsiloxane)/nickel ferrite/carbon nanotube-coated fabric (~ 150 mm s^−1^) [23], and MXene fabric (~ 280 mm s^−1^) [42]. The good air permeability of the coated fabrics could be ascribed to the fact that a large number of the pores among the intertwined fibers (Fig. 2c) and adjacent yarns (Fig. S1b) were not blocked after PPy6@POTS coating. The good flexibility, breathability, and lightweight of PPy6@POTS guarantee satisfactory wearing comfort for multifunctional clothing. Additionally, the coating of PPy6@POTS is uniformly deposited on each fiber surface, as illustrated by the even distributions of the representative elements nitrogen (from PPy) and fluorine (from POTS) based on energy-dispersive X-ray (EDX) mapping (Fig. 2e). Moreover, the X-ray photoelectron spectroscopy (XPS) spectra of both PPy6 and PPy6@POTS confirmed the existence of nitrogen (from PPy) with different intensities (Figs. 2f and S8), further implying the thin thickness of POTS (as the depth detection limit of XPS is generally in the range of a few nanometers). Such a thin thickness of POTS is critical to simultaneously obtain an uncompromised shielding performance and good wearability (i.e., flexibility and permeability) for the coated fabric.

### Electrical and EMI Shielding Properties of PPyn@POTS Textiles

**Fig. 3:**
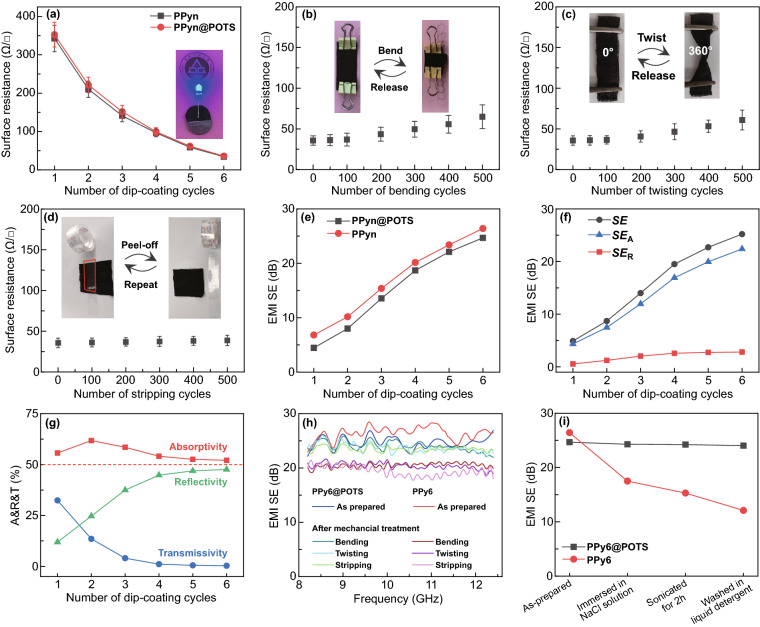
The electrical and EMI shielding properties of PPy*n*@POTS fabrics. **a** Surface resistance of PPy*n*@POTS and PPy fabrics as a function of dip-coating cycles of PPy. The inset is a photograph of a light-up bulb connected with a PPy6@POTS fabric. **b–d** Surface resistance of PPy6@POTS fabric as a function of mechanical treatment cycles, including **b** bending, **c** twisting and **d** stripping. **e** *SE* of PPy*n* and PPy*n*@POTS fabrics as a function of dip-coating cycles. **f** EMI shielding index and **g** absorptivity (*A*), reflectivity (*R*), and transmissivity (*T*) of PPy*n*@POTS fabrics as a function of dip-coating cycles at 10 GHz. **h** *SE* of PPy6@POTS and PPy6 fabrics after bending, twisting, and stripping for 500 cycles. **i** Washing stability of PPy6@POTS and PPy6 fabrics

We first characterized the surface resistance since the electrical conductivity is critical and affects the shielding efficiency. As plotted in Fig. 3a, one cycle of deposition of PPy converted the insulative cotton fabric into a conductive fabric with a surface resistance of 352.7 ± 32.3 Ω □^−1^. This value was continuously reduced to 34.3 ± 6.2 Ω □^−1^ for PPy6. The addition of a thin POTS layer marginally affected the surface resistance of PPy6@POTS (increasing only slightly to 35.7 ± 5.8 Ω □^−1^). Such good conductivity of the PPy6@POTS fabric enabled it to even function as a connecting wire in a circuit to light up a light-emitting diode (Fig. 3a inset). Notably, the introduction of the POTS layer improved the stability of the PPy*n*@POTS fabric’s conductivity to some extent compared to that with only the PPy coating. For instance, the surface resistance of PPy6 increased significantly from 34.3 to 85.7, 87.3, and 89.1 Ω □^−1^ after 500 cycles of bending, twisting, and stripping (Fig. S9), respectively, which was higher than that of the PPy6@POTS fabric under the same treatments (Fig. 3b–d). However, one should note that the surface resistance of the PPy6@POTS fabric also gradually increased under long-term mechanical disturbances. Specifically, the surface resistance increased slightly from 35.7 to 36.9 Ω □^−1^ after 100 bending cycles and subsequently increased to 64.9 Ω □^−1^ after 500 bending cycles (Fig. 3b). A similar surface resistance change pattern was recorded for the successive twisting test, increasing to 60.9 Ω □^−1^ after 500 twisting cycles (Fig. 3c). In addition, mechanical detaching using commercial adhesive tape (~ 5 N stripping force, as shown in Fig. 3d inset) increased the surface resistance to 39.2 Ω □^−1^ after 500 detaching cycles (Fig. 3d). Figure 3b–d shows that the surface resistance of the PPy6@POTS fabric changes little after 100 cycles of bending, twisting, or stripping treatment, and then the surface resistance slowly increases with continuously increasing bending, twisting or stripping cycles. In contrast, the surface resistance of PPy6 fabric without protection coating was always higher than that of PPy6@POTS fabric with POTS protection after the same treatment of mechanical disturbances. For instance, the surface resistance increased from 34.3 to 50.5, 51.9, and 53.5 Ω □^−1^ for PPy6 fabric after 100 cycles of bending, twisting, or stripping treatment (Fig. S9), which is in sharp contrast to PPy6@POTS fabric. Based on these observations, we speculate that POTS could exert appropriate protections for PPy6@POTS fabric under mild mechanical disturbances, especially for the stripping damage. The stable electrical conductivity, even after various mechanical disturbances, possibly originates from the strong interactions among the cotton fibers, PPy and POTS. Specifically, both hydrogen bonding forces (hydroxyl groups from cotton and –NH from PPy) and physical interlocking force between cotton fibers and PPy confer the stable absorption of PPy on fibers [43]. Such hydrogen bonding force and physical interlocking force also exist between PPy and POTS, thus ensuring a compact and stable hybrid coating.

For shielding effectiveness, both PPy*n* and PPy*n*@POTS had a positive correlation with the deposition cycles of PPy, as shown in Fig. 3e. For instance, PPy1 and PPy1@POTS presented average *SE* values of 6.8 and 4.7 dB, respectively, which increased to 26.4 (PPy6) and 24.7 dB (PPy6@POTS) after 6 deposition cycles. Although the shielding performance is not state-of-the-art, the PPy coating is good enough to generate thermal energy by ohmic and dielectric losses under electromagnetic radiation. This thermal effect on the near-instantaneous self-healing of the thin POTS layer is our focus in this work and is discussed in the following sections. Nevertheless, PPy6@POTS already displayed a competitive *SE* compared to the typical commercial requirement of 20 dB [18]. It should also be noted that the PPy layer is the only effective material for EMI shielding for our coated fabric, making its shielding mechanism simpler than those of other reported shielding materials with hybrid components or complex structures. Thus, the discussion or analysis of the self-healing process of POTS (triggered by a PPy layer under electromagnetic wave radiation) is straightforward and requires investigation. It is well known that the shielding mechanism includes reflection, absorption, and multiple reflections. Among them, PPy is a typical absorption-dominant EMI shielding material [21, 44]. Moreover, the porous feature of the fabric substrate enhances the multiple reflections of electromagnetic waves at various surfaces or interfaces in the modified fabric [44]. Based on these two factors, *absorption* is the main shielding mechanism for PPyn@POTS. As plotted in Fig. 3f, *SE*_*A*_ was much larger than *SE*_*R*_. Additionally, the electromagnetic energy coefficients of absorptivity (*A*), reflectivity (*R*), and transmissivity (*T*) were also calculated (*A* = 1 − *R* − *T*) [38] (see Experimental Section). As seen in Fig. 3g, A was always higher than *R* and *T* and crossed the 50% benchmark. Therefore, such high absorptivity means that the electromagnetic energy is mainly absorbed by the PPy layer and then converted into thermal energy, which is ultimately dissipated into the surroundings. This adsorption-dominant feature could eliminate the secondary EMI pollution caused by reflected electromagnetic waves. More importantly, this feature would enable the coated fabric to convert the majority of electromagnetic energy into thermal energy under intense irradiation and rapidly increase the temperature of the fabric within a short period, which is vital for the following discussed superfast self-healing process.

The shielding performance stability of PPy6@POTS in the range of 8.2–12.4 GHz when subjected to mechanical bending, twisting, and stripping was studied, as displayed in Fig. 3h. Similar to the surface resistance change behavior discussed earlier, the stability was enhanced by introducing the thin POTS layer. Briefly, the average *SE* decreased for PPy6 from 26.4 to 20.5, 20.4, and 19.4 dB after 500 cycles of bending, twisting, and stripping, respectively. However, the average *SE* of the PPy6@POTS fabric (original 24.7 dB) slightly decreased after 500 cycles of bending (to 24.0 dB), twisting (to 23.7 dB) and stripping (to 23.5 dB). Furthermore, the EMI *SE* of PPy6@POTS demonstrated relatively good stability when subjected to a salt solution for a short period. For instance, the immersion of PPy6@POTS fabric into a NaCl solution for 96 h resulted in only a slight change in the average *SE* from 24.7 to 24.3 dB (Fig. 3i). Other conditions, such as sonication in DI water for 2 h or regular machine washing with liquid detergent for 45 min, did not apparently deteriorate the shielding performance. In contrast, the average *SE* of PPy6 without the POTS protection layer deteriorated severely from 26.4 to 17.5, 15.3, and 12.1 dB after NaCl solution washing (96 h), DI water sonication (2 h), and liquid detergent washing (45 min), respectively (Figs. 3i and S10). Such stability magnified the benefit of using the hydrophobic POTS layer to protect the EMI shielding property in various harsh environments.

### Protection Effectiveness of POTS Subject to Long-term Applications

**Fig. 4:**
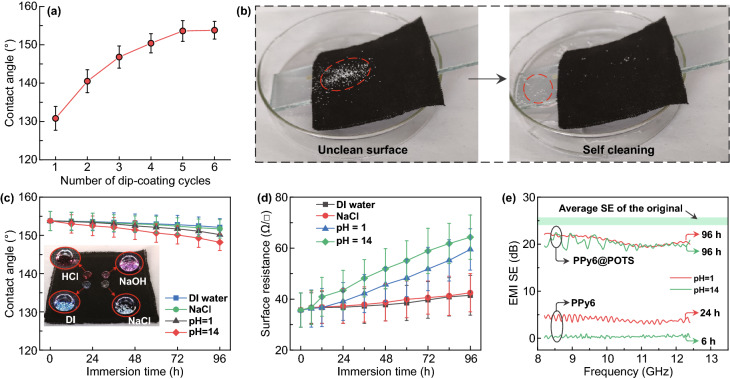
Protections of POTS on multifunctional PPy*n*@POTS fabrics. **a** Water contact angle of PPy*n*@POTS fabrics. **b** Self-cleaning process of PPy6@POTS fabric. Dashed red cycles indicate the position of dust. **c** Water contact angle and **d** surface resistance of PPy6@POTS after immersion in DI water, NaCl, HCl (pH 1) and NaOH (pH 14) solutions for various times. The inset in **c** shows droplets of DI water, NaCl, HCl and NaOH on a PPy6@POTS fabric. An air cushion between the liquid and the fabric surface could be observed. **e** *SE* of PPy6@POTS and PPy6 fabrics after immersion in aqueous HCl (pH 1) and NaOH (pH 14)

To deliver a detailed understanding of POTS’s protective effect on the EMI shielding performance, the CA of PPy*n*@POTS fabric with 1–6 deposition cycles of PPy was first characterized. The hydrophilic surface of PPy6 was revealed by a CA of 71.3° (Fig. S11a). However, after introducing the POTS layer (a low-surface-energy material with perfluorinated groups, Fig. S12), fabrics became hydrophobic with a CA of 130.8° for PPy1@POTS (Fig. 4a). The hydrophobicity was further enhanced as the deposition cycles of PPy increased, reaching 153.8° for PPy6@POTS (Figs. 4a and S11b) because of the increasing roughness of the fiber surface (Fig. 2a–c and S2, S3). This high CA made the PPy6@POTS fabric superhydrophobic with a self-cleaning capability. As a demonstration shown in Fig. 4b, the stained surface (chalk dust) of PPy6@POTS fabric can be easily and quickly cleaned by water flows.

Additionally, the stability of the superhydrophobic surface by POTS coating was further evaluated by immersing the PPy6@POTS fabrics in different liquid solutions (i.e., DI water, NaCl (1 M), HCl (pH 1), and NaOH (pH 14), which represent the neutral environment, human sweat, and harsh acidic and basic environments, respectively). The superhydrophobicity of the POTS-coated surface allowed a layer of air cushion between the fabric and surrounding solution (known as the formation of Cassie–Baxter state [31, 45], Fig. 4c inset), reducing the possible interactions between the solution and the coating. As a result, the overall stability of PPy6@POTS was much better than that of PPy6, including CA, surface resistance, and EMI *SE* (Fig. 4c–e and S13). Nevertheless, we noticed that the stable properties of PPy6@POTS gradually degraded to some extent when extending the immersion time. Specifically, the CA decreased for all solution cases with a prolonged immersion time (the worst for NaOH (pH 14) case from 153.8° to 148.2°, Fig. 4c), possibly due to the desorption of POTS molecules [46]. The protection of POTS on the surface resistance retention was even worse (Fig. 4d). After 96 h of immersion, the surface resistance increased from 35.7 to 41.5 Ω □^−1^ for DI water and 42.3 Ω □^−1^ for aqueous NaCl. In acidic and basic environments, the surface resistance was relatively stable after the first 12 h of immersion (36.7 Ω □^−1^ for pH 1 and 36.9 Ω □^−1^ for pH 14) and increased to 59.5 Ω □^−1^ (pH 1) and 64.3 Ω □^−1^ (pH 14) after 96 h of immersion, respectively. The faster resistance increase in the strong alkaline environment after more than 12 h of immersion may originate from the hydrolyzation between hydrolysable groups of POTS and hydroxyl groups of the alkaline [47], leading to the decreased protection performance. This surface resistance increase deteriorated the shielding performance from 24.7 to 20.8 dB for pH 1 and 20.2 dB for pH 14, as shown in Fig. 4e. These results indicated that even with the POTS protection layer, the shielding property of PPy6@POTS was still subject to long-time water, salt, acid, or base solution invasion and even mechanical disturbance (Figs. S14 and S15). Therefore, the durability and robustness of the protection coating should be further improved to achieve durable shielding performance for long-term applications. We resolve this issue by introducing the instantaneously self-healing feature of the POTS layer via microwave heating.

### Near-Instantaneously Self-healing of POTS for Durable Shielding Performance

**Fig. 5:**
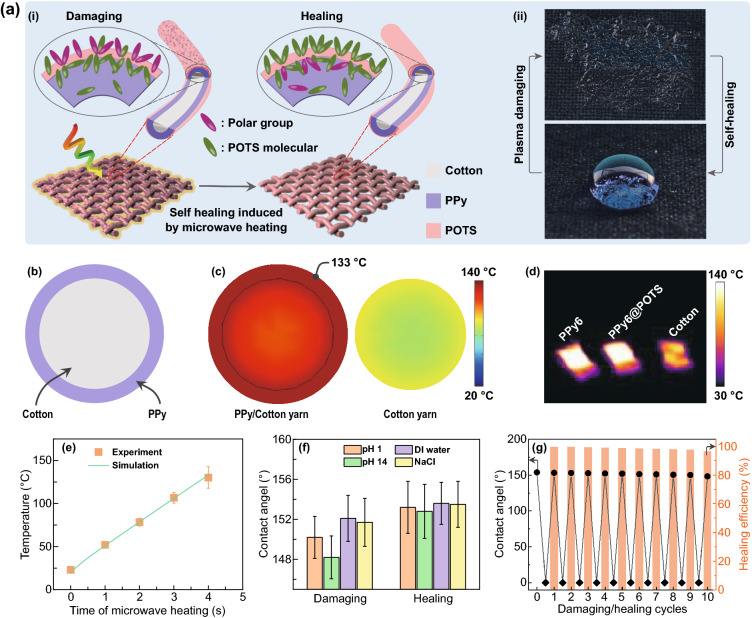
Near-instantaneous self-healing of PPy*n*@POTS fabrics via microwave heating. **a** Schematic of the POTS self-healing mechanism on a PPy*n*@POTS fabric (i) and the instant recovered water contact angle after plasma damage and self-healing via microwave heating (ii). **b** Cross-sectional geometry of a simplified PPy/cotton yarn model. **c** Simulated temperature profiles of a PPy/cotton yarn and bare cotton yarn after 4 s of microwave heating. **d** Infrared thermal image of raw, PPy6, and PPy6@POTS fabrics after microwave heating for 4 s. **e** Surface temperature plot of both experimental and simulated results. **f** Water contact angle of PPy6@POTS fabrics after damage by 96 h of immersion in different solutions and the corresponding healed water contact angle by microwave heating. **g** Water contact angle and healing efficiency of PPy6@POTS fabric with excellent stability after 10 damaging/healing cycles

When the hydrophobic surface of POTS was chemically destroyed, oxygen-containing polar groups appeared on the surface, making it hydrophilic with a decreased CA (Fig. 5a, i). Under natural conditions, the self-healing of POTS is very slow. Thus, it was reported that heat (temperature control) could facilitate the restoration of the superhydrophobicity by accelerating the migration of intact POTS molecules to the coating surface by embedding polar groups after damage [34, 40, 48]. As a triggering stimulus, heat (from various thermal sources) is commonly applied for self-healing due to its easy application. However, the corresponding healing time ranges from several minutes to hours because of the relatively slow molecular migration speed [49]. By taking advantage of the microwave heating effect of PPy, in this study, the thermal energy needed for self-healing of POTS (~ 130 ℃) was achieved within a few seconds (4 s) through intense microwave irradiation (using a household microwave oven), which is at least one magnitude faster than the best results in the literature (Table S1) [50].

As discussed earlier, PPy, as an EMI shielding material, can absorb electromagnetic waves from the environment and convert them into low-level thermal energy via ohmic and dielectric losses [9, 51]. Furthermore, a tremendous amount of heat can be generated by PPy if intense microwave irradiation occurs. We simulated the heating effect of PPy6@POTS using a simplified PPy/cotton yarn model to prove this speculation. The cross section of PPy/cotton yarn is schematically illustrated in Fig. 5b, where PPy was set to 20 μm thick, and the diameter of cotton yarn was fixed at 250 μm (Experimental Section and Fig. S1). The microwave heating effect of POTS was omitted, as its thickness was negligible (Figs. 2b, c, f and S3) compared to the dimensions of the PPy coating and cotton yarn. Figure 5c presents the temperature profile of the yarn cross sections after 4 s of microwave irradiation. A significant amount of heat was generated by PPy, resulting in a surface temperature as high as 133 ℃ (still lower than the decomposition temperature of PPy (~ 200 ℃, Fig. S16). Compared to the bare cotton yarn, the high temperature of PPy/cotton yarn was ascribed to the heat transfer from the irradiated PPy. Experimentally, both PPy6 and PPy6@POTS fabric were heated quickly to ~ 130 ℃ after 4 s using a household microwave oven (Fig. 5d), which was in good agreement with the simulated temperature results (Fig. 5e). This further verified both the microwave heating effect of PPy and the solidification of the simplified PPy/cotton yarn model.

Given this instant heat (4 s) from PPy via microwave heating, we further investigated the self-healing of POTS that was damaged by long-time immersion (96 h) in various solutions (DI water, aqueous NaCl, HCl (pH 1), and NaOH (pH 14)). As shown in Fig. 5f, the CAs of PPy6@POTS fabrics completely recovered from 150.2° (HCl), 148.2° (NaOH), 152.1° (DI water), and 151.7° (NaCl) to 153.2°, 152.8°, 153.6°, and 153.5°, respectively. This near-instantaneous (4 s) self-healing property can be ascribed to the following two reasons. First, the strong interactions between PPy and incident electromagnetic waves enable the microwave energy to be mainly converted to thermal energy (~ 50%), generating sufficiently high temperatures in a few seconds. Second, microwaves from oven heating radiate and penetrate the fabric three-dimensionally, which results in faster and more uniform temperature distribution, thus significantly shortening the heat exchange time within the whole coating layer compared to that of the traditional heat transfer method [49, 52]. Therefore, the rapidly and uniformly heated PPy layer dramatically accelerates the migration of POTS molecules to the surface and thus regenerates the protective properties of the coating layer.

To further demonstrate the effectiveness of the microwave heating method for near-instantaneous self-healing, severe oxygen plasma etching (30 s) was intentionally applied to the PPy6@POTS fabric. Notably, the damaged surface was nearly instantaneously restored (recovered water CA) by just 4 s of microwave irradiation (Fig. 5a–ii). This damaging/healing process was repeatable (Fig. 5g), demonstrating the effectiveness and robustness of the microwave heating method. To evaluate the self-healing ability, healing efficiency (%) was introduced and was calculated as the CA ratio:

$$Healing\; \, efficiency = CA_{{{\text{healed}}}} /CA_{{{\text{original}}}} \times 100\%$$

where *CA*_original_ and *CA*_healed_ are the water contact angles of the original samples and healed samples (by the microwave heating method), respectively. As shown in Fig. 5g, the healing efficiency of PPy6@POTS fabrics is 99.0% after 4 healing cycles, and it remains at 96.4% even after 10 damaging/healing cycles. The high healing efficiency further verified the reliability of the self-healing process using the microwave heating method.

**Fig. 6:**
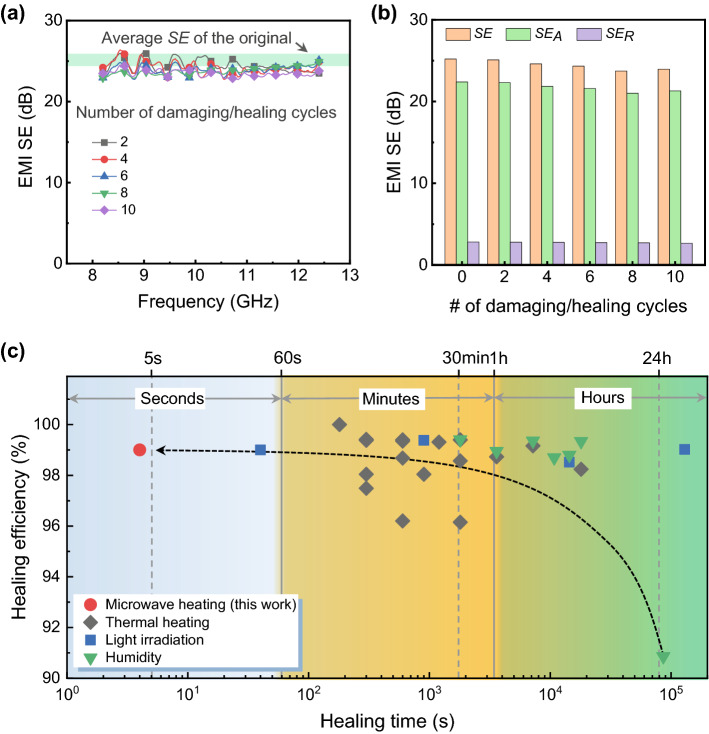
Durable EMI shielding performance and comparison between various self-healing methods. **a** *SE* curves and **b** the corresponding *SE*_*R*_ and *SE*_*A*_ values at 10 GHz of PPy6@POTS fabric after various damaging/healing cycles. The shaded green area in **a**, as a guide for the eye, is the average *SE* of the original sample. **c** Comparison of healing efficiency and healing time of the PPy6@POTS fabric via the microwave heating approach with others reported in the literature. The microwave heating method stands out among the many other approaches due to its superfast healing process and comparable healing efficiency

Instant self-healing is not only crucial for restoring the superhydrophobicity of POTS but also critical for promptly protecting the underlying EMI shielding materials when subject to physical or chemical disturbance. The healing capability could broaden the applications to many other fields [53, 54]. Figure 6a presents the excellent, resilient, and stable shielding performance of the PPy6@POTS fabric after 10 cycles of severe damage (plasma etching) followed by self-healing (microwave heating). However, without the self-healing process, the shielding efficiency of the PPy6@POTS fabric seriously deteriorated after plasma etching and decreased to ~ 10 dB after 10 cycles of damage (Fig. S17). This could be explained by the decreased surface resistance of the PPy6@POTS fabric (Fig. S18), where the conductive PPy network was destroyed by plasma damage if the POTS layer was not promptly healed. Moreover, the shielding mechanism was kept the same after the cyclical damaging/healing process. As displayed in Fig. 6b, the *SE*_*A*_ and *SE*_*R*_ values of the PPy6@POTS fabric were almost unchanged after 10 cycles, indicating the well-retained structures and properties of the PPy layer assisted by the instantaneously self-healing process (Fig. S18). Additionally, when compared to other self-healing approaches, such as humidity control [31], photothermal effects [50], or direct heating [34], the self-healing performance of POTS using our proposed approach via microwave heating is significantly advantageous in terms of healing time (as quick as 4 s), which is at least one magnitude faster and features a decent healing efficiency of ~ 99.0% (Fig. 6c and Table S1). Therefore, such a prompt and near-instantaneously self-healing feature of POTS with excellent protection capability in various harsh environments could expand the potential of EMI shielding materials for applications where stability and durability are highly weighted.

## Conclusions

In summary, we reported a superfast yet simple approach via microwave heating to extend the service lifespan of EMI shielding materials, achieving stable and durable performance, even in harsh environments. The superhydrophobic surface formed by coating a thin POTS layer on PPy-modified fabrics not only provided a self-cleaning feature (water contact angle > 150°) but also served as a protective layer to maintain good conductivity (~ 35.7 Ω □^−1^) and shielding performance (~ 24.7 dB) in DI water, aqueous NaCl, and even corrosive acidic and basic solutions. Additionally, after damage, the protection capability of the POTS layer can be repeatedly self-healed via microwave heating within just 4 s. This near-instantaneously self-healing feature of the POTS layer enables its long-term protection of the conductivity and shielding efficiency of PPy6@POTS fabrics. More importantly, this approach could be expanded to other EMI shielding material systems with different protection layers as long as heat is a triggering stimulus for healing. We envision that the microwave heating approach proposed in this work will provide insights into EMI shielding material preparations for achieving durable performance and could have significant influence and potential applications in the field of portable and wearable devices.

## Supplementary Information

Below is the link to the electronic supplementary material.

Supplementary file 1 (PDF 960 KB)
