# Supplementary material for: Near-Instantaneously Self-Healing Coating toward Stable and Durable Electromagnetic Interference Shielding
Source: Nanomicro Lett. 2021 Sep 8;13:190. doi: 10.1007/s40820-021-00709-0 (PMC8426454; doi:10.1007/s40820-021-00709-0)
Supplement: Supplementary file 1 — (PDF 960 KB) [file 40820_2021_709_MOESM1_ESM.pdf]

Supporting Information for

Near-Instantaneously Self-Healing Coating towards Stable and Durable Electromagnetic Interference Shielding

Lihua Zou[1, 4], Chuntao Lan[3], Songlin Zhang[2, *], Xianhong Zheng[1], Zhenzhen Xu[1, *], Changlong Li[1], Li Yang[1], Fangtao Ruan[1], Swee Ching Tan[2, *]

[1] Anhui Province International Cooperation Research Center of Textile Structure Composite Materials, Anhui Polytechnic University, Anhui, Wuhu 241000, P. R. China

[2] Department of Materials Science and Engineering, National University of Singapore, 9 Engineering Drive 1, Singapore, 117575 Singapore

[3] Key Laboratory of Eco-Textiles, Ministry of Education, Jiangnan University, Wuxi, Jiangsu 214122, P. R. China

[4] Department of Mechanical Engineering, University of Delaware, Newark, DE 19716, USA

*Corresponding authors. E-mail: msezs@nus.edu.sg (Songlin Zhang), xuzhenzhen@ahpu.edu.cn (Zhenzhen Xu), msetansc@nus.edu.sg (Swee Ching Tan)

Supplementary Tables and Figures

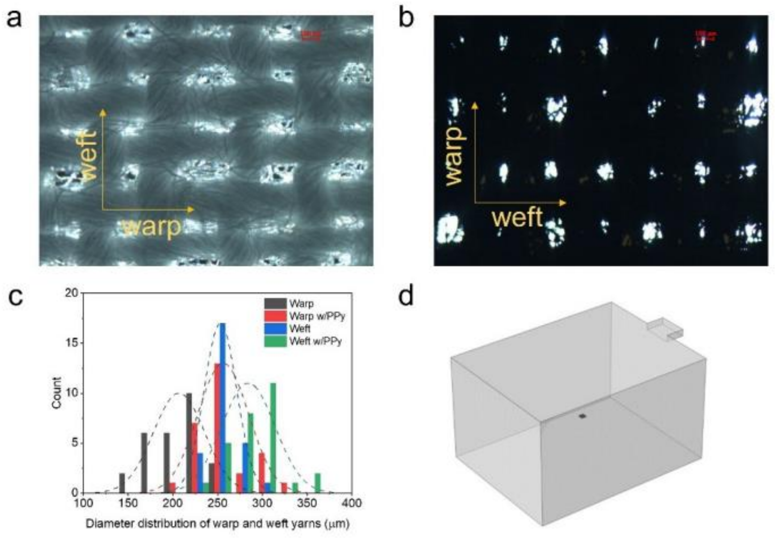

Fig. S1 (**a**) The optical images of the pristine cotton fabric, and (**b**) the fabric coated with PPy6@POTS. The scale bar is 100 μm. (**c**) The histogram of cotton yarn's diameter (both weft and warp) with and without PPy6@POTS coating. (**d**) The setup of COMSOL simulation using a household microwave oven where the PPy/cotton yarn model was placed at the bottom plate's center.

The geometry of PPy/cotton yarn was estimated based on the diameters of both weft and warp yarns before (Fig. S1a) and after (Fig. S1b) coating. As indicated in Fig. S1c, the pristine fabric's average diameters of both warp and weft yarns are 206.9 and 253.1 µm, respectively. And the PPy6@POTS coated cotton yarns' average diameters of both warp and weft yarn are 256.3 and 284.3 µm, respectively. Thus, the thickness of PPy@POTS coating was roughly around 49.4 (warp) and 31.2 (weft) µm. Based on this information, a representative diameter of PPy/cotton yarn was set as 290 µm, where cotton yarn was 250 µm in diameter, and the thickness of PPy coating was 20 µm.

The COMSOL parameters applied for the simulation setup in Fig. S1d were as follow:

Microwave oven geometryOven width: 482 mm

Oven depth: 368 mm

Oven height: 282 mm

Waveguide geometry

Waveguide width: 50 mm

Waveguide depth: 78 mm

Waveguide height: 18 mm

Initial values in the model

Electric field: 0 V/m

Initial temperature: 20 °C

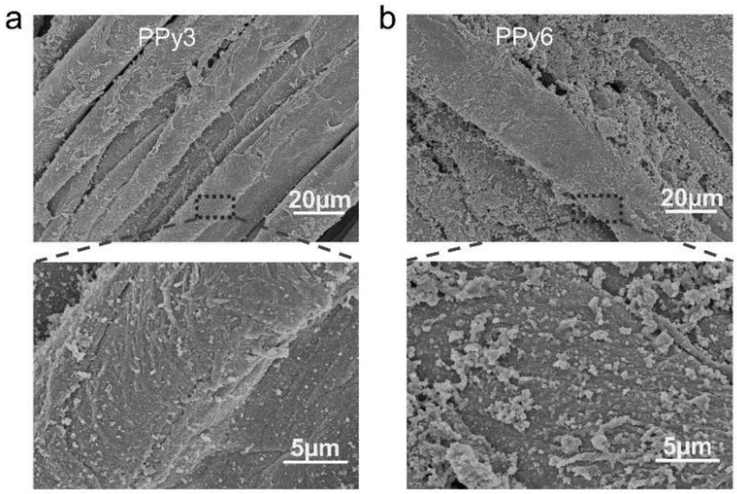

Fig. S2 SEM images of PPy coated cotton fabrics with various PPy deposition cycles (**a**) three times, (**b**) six times, and their enlarged SEM images. PPy nanoparticle uniformly coated onto the cotton fiber. Besides, the PPy loading onto cotton fiber increased with the PPy deposition, which also increased the roughness of the coated fabric.

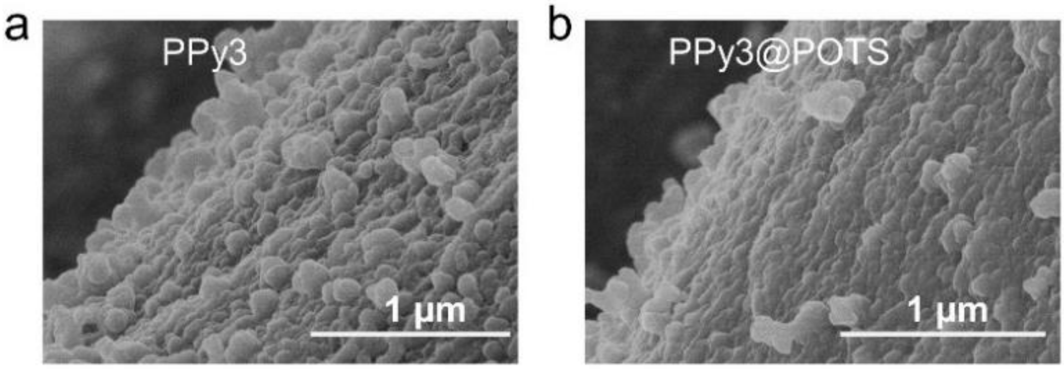

Fig. S3 SEM images of (a) PPy3 cotton fiber. The PPy nanoparticles were densely coated onto the cotton fiber. (b) PPy3@POTS cotton fiber. After coating a POTS layer, the PPy nanoparticle is still clear to see, which means that the POTS layer is so thin that the PPy nanoparticle only slightly been covered.

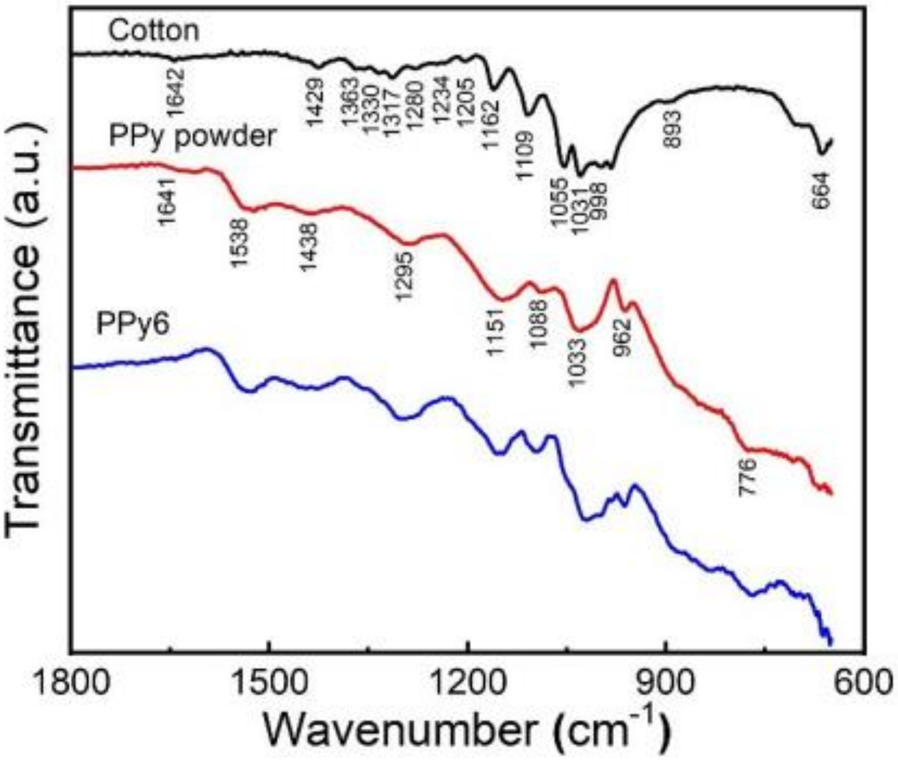

Fig. S4 FT-IR spectra of PPy powder, pristine cotton (Cotton) and PPy coated cotton (PPy6). On PPy6, the superimposed bands of PPy powder and cotton were recorded at 1537 cm^{-1} (C=C stretching), 1437 cm^{-1} (C-C stretching), 1295 cm^{-1} (C-N stretching), 1151 cm^{-1} (C=N stretching), 1088 cm^{-1} (N-H stretching), and 1033 cm^{-1} (N-H wagging) [1], which proves PPy was successfully deposited onto cotton fabric.

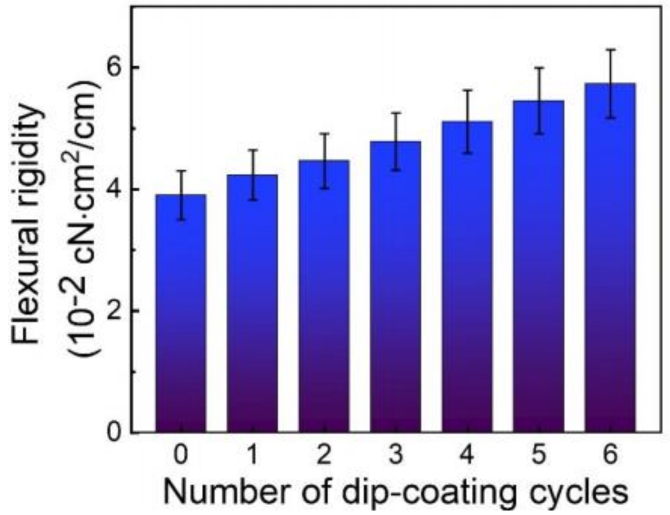

Fig. S5 Flexural rigidity of PPyn@POTS fabrics with a number of dip-coating cycles. The flexural rigidity of PPyn@POTS fabrics increased with the number of dip-coating cycles. Specifically, the flexural of PPy6@POTS fabric was 5.73 cN cm^2 cm^{-1}, increased from 3.9 cN cm^2 cm^{-1} of the raw fabric. However, it still kept good flexibility, as discussed in Fig. 2

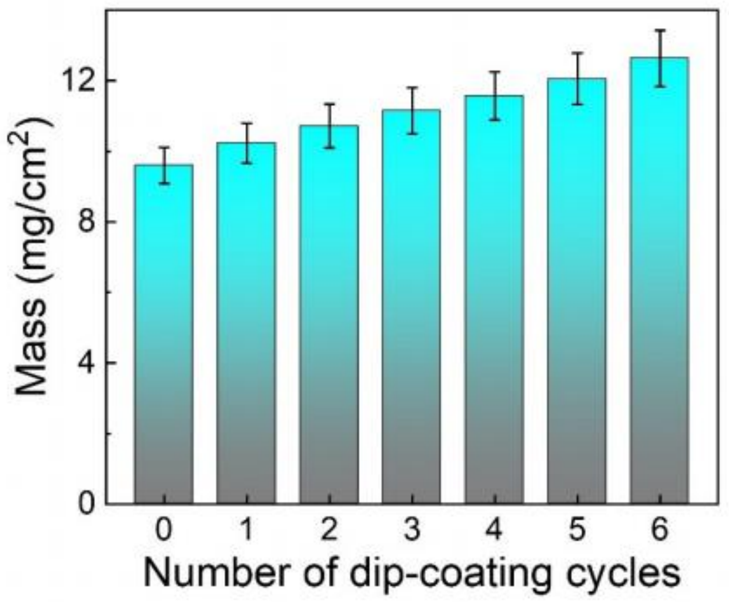

Fig. S6 Area mass density of PPyn@POTS fabrics with the number of dip-coating cycles. The mass of PPyn@POTS fabrics increased with the number of PPy dip-coating cycles. Specifically, the mass of raw fabric is 9.600 mg cm^{-2}, and increased to 12.633 mg cm^{-2} after six times dip coating.

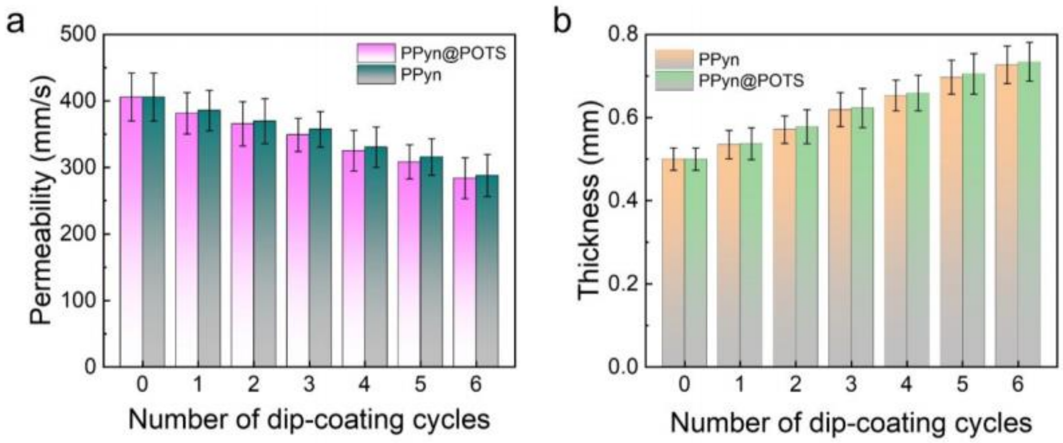

Fig. S7 (**a**) Air permeability and (**b**) the thickness of PPyn@POTS and PPyn fabrics with a number of dip-coating cycles. The air permeability of PPyn@POTS fabrics decreased with the number of dip-coating cycles, which is ascribed to the blocking of pores between fibers or yarns. There is only slightly change of fabric thickness after deposition of POTS.

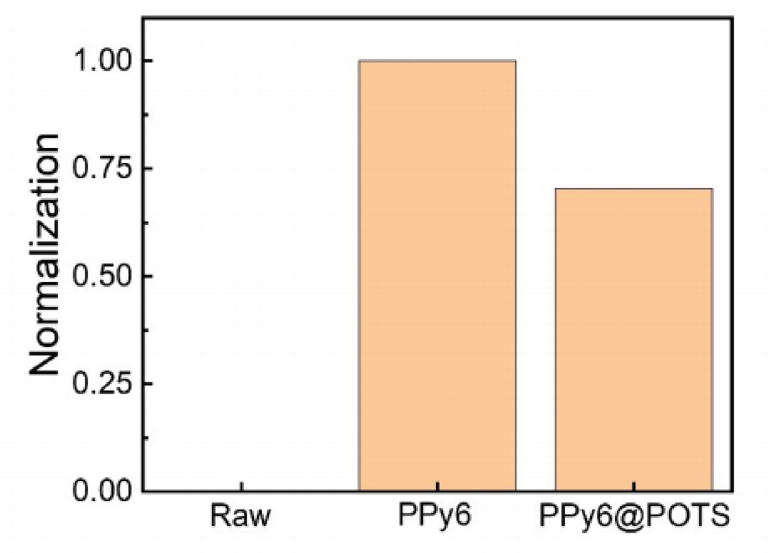

Fig. S8 Intensity comparison of N 1s from the XPS spectrum for the Raw cotton, PPy6 and PPy6@POTS fabric. To compare the change of nitrogen after POTS deposition, the three samples' nitrogen (N) intensity was normalized. As shown in Fig. S7, the PPy6 is 1.00, and the Raw fabric is 0 (there is no nitrogen in raw fabric), and the PPy6@POTS is 0.704. This result indicates that the POTS layer is very thin due to the depth detection limit of XPS (in the range of a few nanometers).

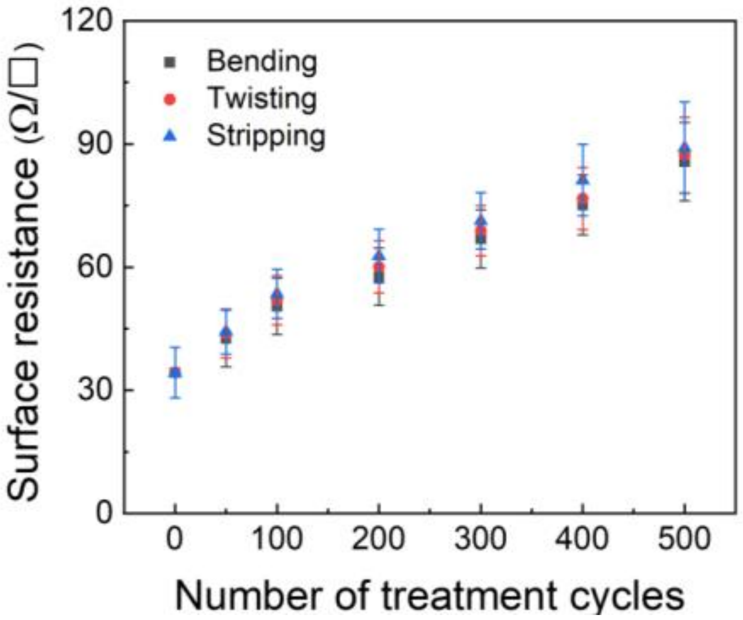

Fig. S9 Surface resistance of PPy6 fabric as a function of bending, twisting and stripping treatment cycles. Without the POTS protection, the conductivity of the PPy6 dramatically decreased after mechanical treatment.

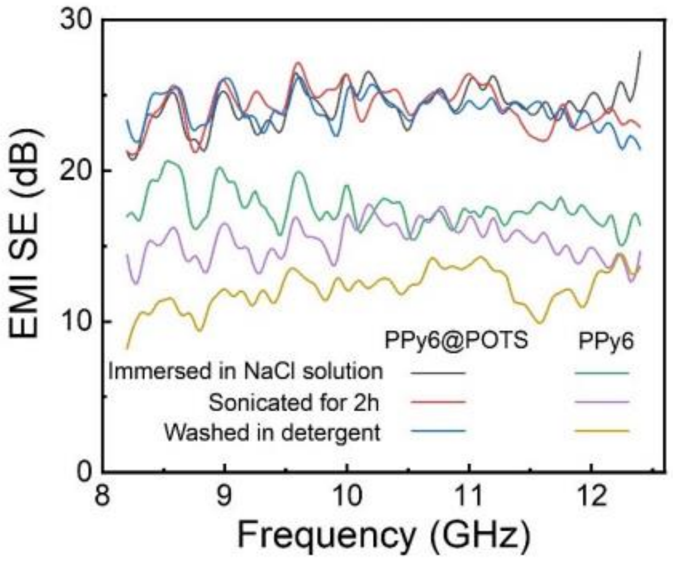

Fig. S10 *SE* curves of PPy6@POTS and PPy6 after different washing in the frequency range of 8.2–12.4 GHz. The EMI *SE* of PPy6@POTS still maintains higher than 20 dB after immersed in NaCl solution for 96 h, sonicated for 2 h and washed in detergent for 45 min. In contrast, the EMI *SE* of PPy6 gradually decreased after different washing treatment, and the average EMI *SE* of PPy6 decreased from 26.4 dB to 17.5, 15. 3 and 12.1 dB after immersed in NaCl for 96 h, sonicated for 2 h and washed in detergent for 45 min, respectively.

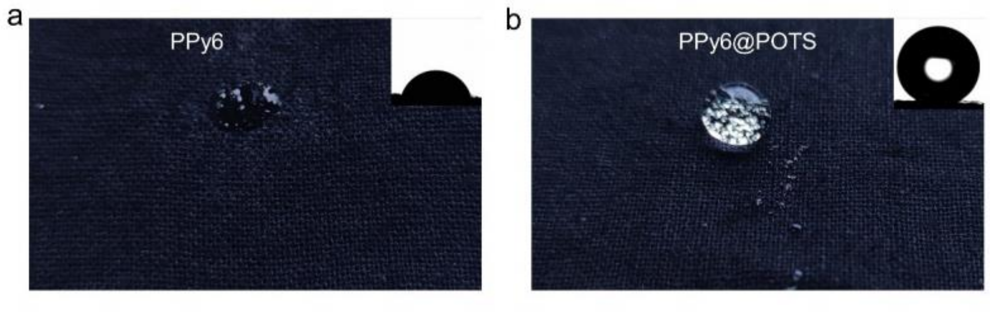

Fig. S11 Photograph of (a) PPy6 with a drop of water on the surface. The inset shows that PPy6 absorbed the water droplet due to the hydrophilic character, whose water contact angle is 71.3°. Photograph of (b) PPy6@POTS fabric after drop a water droplet. After POTS deposition, the PPy6@POTS became superhydrophobic, whose water contact angle is 153.8° (the inset).

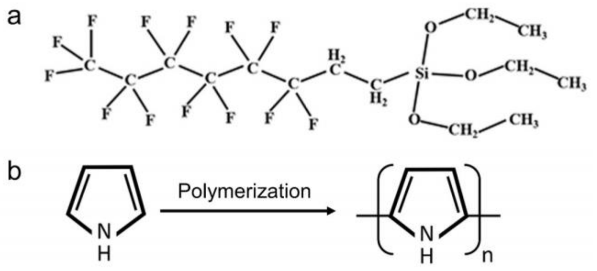

Fig. S12 (**a**) Chemical structure of POTS molecular. The existence of the F element is beneficial for lowing the surface energy of POTS coating. (**b**) Molecular structure of Py and PPy. Polypyrrole (PPy) was prepared by polymerization of the monomer (Py) with $FeCl_3$. From the molecular structure of PPy and POTS, it was evident that the N element comes from PPy, and the F element comes from POTS only, which is in good agreement with the XPS result (Fig. 2f).

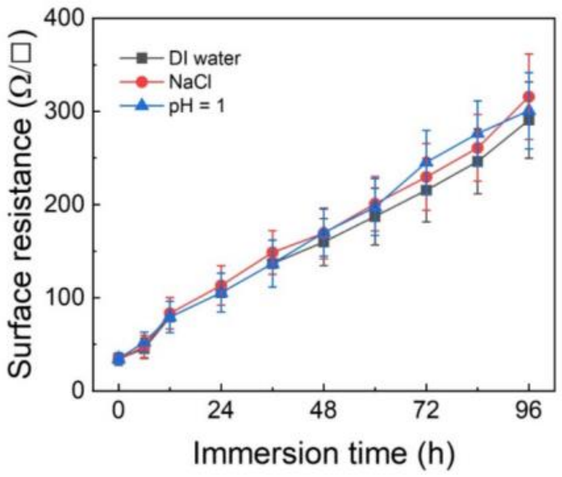

Fig. S13 Surface resistance of PPy6 after immersing in DI water, NaCl solution and HCl (pH = 1) solution for various time. The conductivity of PPy6 was seriously destructed for all solution conditions mentioned above (surface resistance was

increased beyond 290 Ω □$^{-1}$). Even worse, the surface resistance of PPy6 was increased to infinite after only 1 h immersion in a base environment (pH = 14).

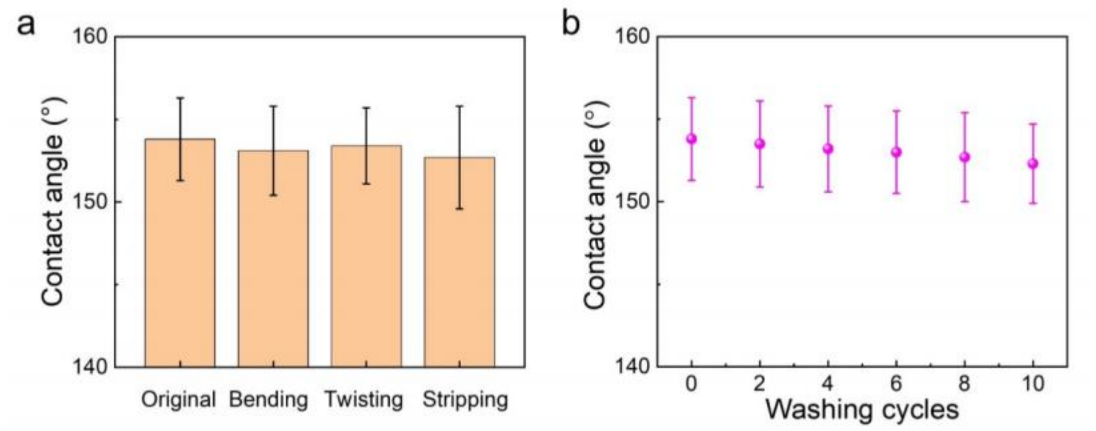

Fig. S14 Water contact angle of PPy6@POTS fabric with (**a**) 500 cycles bending, twisting and stripping and (**b**) various washing cycles. The CA of PPy6@POTS fabric decreased from 153.8° to 153.1°, 153.4° and 152.7° after 500 cycles of bending, twisting and stripping, respectively. After 10 washing cycles the CA of PPy6@POTS fabric decreased to 152.3°.

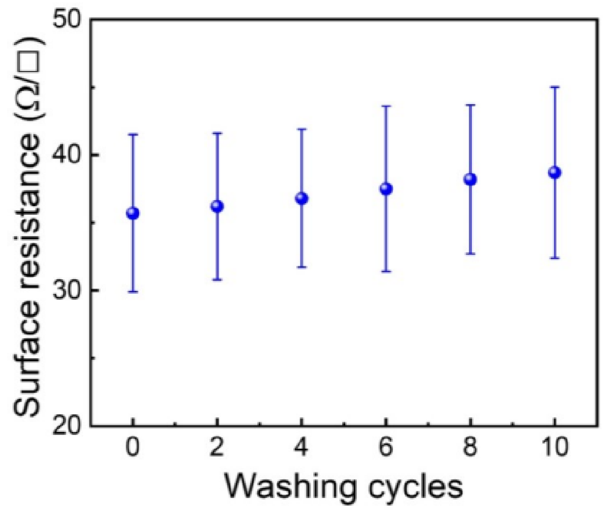

Fig. S15 Surface resistance of PPy6@POTS fabric with various washing cycles. The surface resistance of PPy6@POTS fabric increased to 38.7 Ω □$^{-1}$ after 10 cycles of washing.

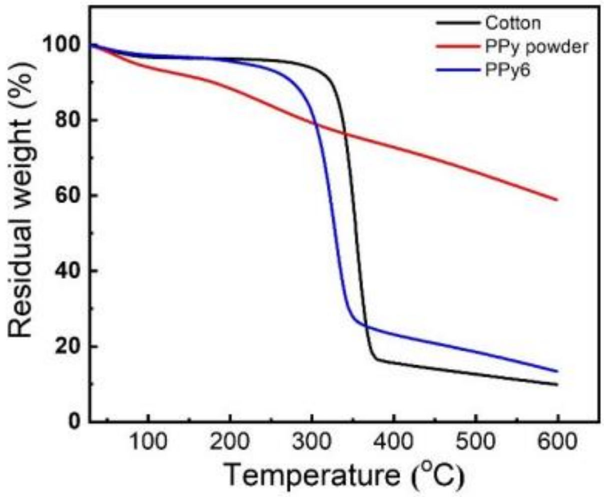

Fig. S16 TG curve of cotton, PPy powder and PPy6 fabric. The TG traces of PPy powder showed a continuous mass loss in the entire temperature range with 41.1% weight loss. In contrast, the TG traces of Cotton and PPy-cotton show a systematic and stepwise weight loss, each corresponding to the loss of particular species. The first small weight loss, around 120 °C, is attributed to the evaporation of water molecules or any other volatile moieties. Afterward, cotton displays good thermal stability, up to 300 °C. After that, the observed degradation of cotton was marked by an abrupt weight loss of 83.1%. On the other hand, PPy6 shows an additional (apart from the loss step of Cotton) weight loss at 200 °C due to degradation of the coated PPy phase. Thus, it is reasonable to speculate that the combination of PPy with cotton increase the thermal stability of PPy coated fabric, which provides a prerequisite for healing through microwave treatment of PPyn@POTS coated fabric.

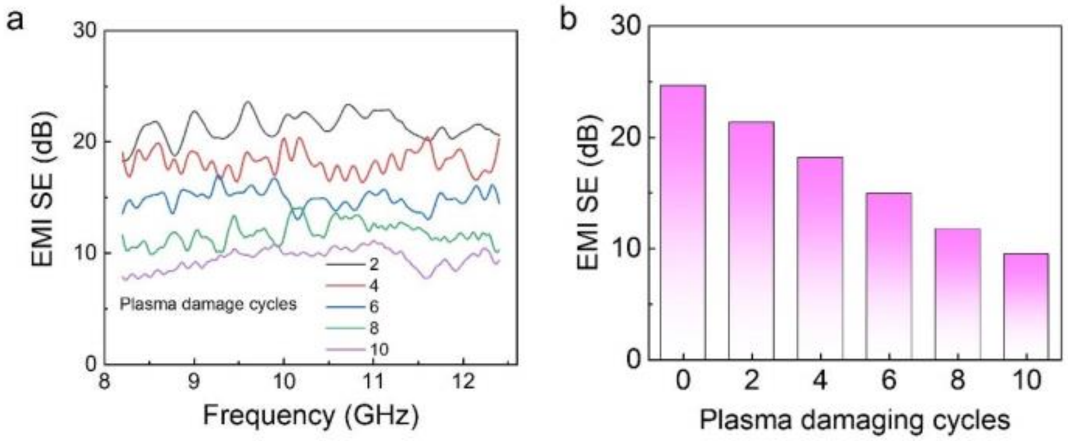

Fig. S17 (**a**) EMI *SE* over the X band and (**b**) Average EMI *SE* of PPy6@POTS fabrics with the plasma treatment times without healing process. The shielding performance of the fabric was continuously decreased after the repeated plasma treatment because of the destruction of the conductive network, which is confirmed by Fig. S16.

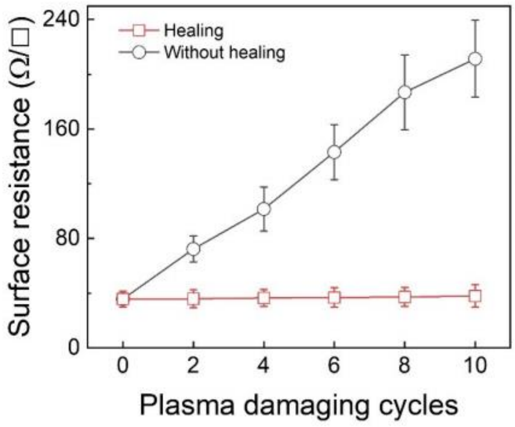

Fig. S18 Surface resistance PPy6@POTS fabric with and without healing process after different plasma treatment times. The surface resistance of PPy6@POTS keeps almost constant at 37.9 Ω^{-1} after 10 times of plasma/healing treatment. Whereas the surface resistance of PPy6@POTS increases sharply to 211.4 Ω $\square^{-1}$ after 10 times of plasma treatment without the healing process.

Table S1 Summary of the self-healing materials reported in the literature

Method	Condition	Time (s)	original	Healed	Efficiency	Ref
NIR [a] light	90 °C	40	158°	156.5°	99% [b]	[2]
Sun light	49 °C	900	161°	160°	99.38%	[3]
Sun light	70 °C	300	159.5°	155.5°	97.49%	[4]
Electrothermal	135 °C	600	167°	166°	99.4% [b]	[5]
UV [c] irradiation	60 °C	14400	155.3°	153°	98.52% [b]	[6]
UV irradiation	-	129600	152.8°	151.3°	99.02%	[7]
Heating	135 °C	180	171°	171°	100%	[8]
Heating	100 °C	300	153°	150°	98.04%	[9]
Heating	130 °C	300	172°	171°	99.42%	[10]
Heating	130 °C	300	161°	160°	99.38%	[11]
Heating	135 °C	600	151°	149°	98.68%	[12]
Heating	130 °C	600	154°	153°	99.35%	[13]
Heating	150 °C	600	158°	152°	96.20% [b]	[14]
Heating	80 °C	900	153°	150°	98.04% [b]	[15]
Heating	80 °C	1200	157°	130°	82.8%	[16]
Heating	80 °C	1200	145°	144°	99.31%	[17]
Heating	40 °C	1800	161.3°	159°	98.57% [b]	[18]
Heating	80 °C	1800	167°	166°	99.40%	[19]
Heating	100 °C	1800	156°	150°	96.15% [b]	[20]
Heating	80 °C	3600	158°	156°	98.73% [b]	[21]
Heating	60 °C	7200	154.2°	152.9°	99.16%	[22]
Heating	70 °C	18000	170°	167°	98.24%	[23]
Moisture	55% RH [d]	1800	169°	168°	99.41%	[24]
Moisture	35% RH	3600	160°	158°	98.95% [b]	[25]
Moisture	84% RH	7200	157°	156°	99.36%	[26]
Moisture	55% RH	10800	156°	154°	98.72%	[27]
Moisture	40% RH	14400	166°	164°	98.80 % [b]	[28]
Moisture	50% RH	18000	152°	151°	99.34% [b]	[29]
Microwave heating	130 °C	4	153.8°	152.3°	99.0%	This work

Notes: [a] NIR: near-infrared ray, [b] This efficiency data was calculated using contact angle information from the reference, [c] UV: ultraviolet light, [d] RH: relative humidity, [−] not mentioned in the reference

Table S2 PPy and cotton properties for modeling

	PPy	Cotton	Refs
Electrical conductivity (S m^{-1})	1500	0	[30]
Thermal conductivity (W m^{-1} K^{-1})	50	0.05	[30][31]
Heat capacity Cp (J kg^{-1} K^{-1})	1400	1300	[30][32]
Permittivity	4 - 0.85×j	2.231 - 0.181×j	[30][33]
Relative permeability	1	1	
Density (kg m^{-3})	1150	1540	[30][34]

Supplementary References

[S1] Z. Stempien, T. Rybicki, E. Rybicki, M. Kozanecki, M.I. Szynkowska, In-situ deposition of polyaniline and polypyrrole electroconductive layers on textile surfaces by the reactive ink-jet printing technique. Synth. Met. **202**, 49-62 (2015). https://doi.org/10.1016/j.synthmet.2015.01.027

[S2] Y. Liu, X. Pei, Z. Liu, B. Yu, P. Yan et al., Accelerating the healing of superhydrophobicity through photothermogenesis. J. Mater. Chem. A **3**, 17074-17079 (2015). https://doi.org/10.1039/C5TA04252F

[S3] J. Zhang, J. Zhao, W. Qu, Z. Wang, Fabrication of superhydrophobic fabrics with outstanding self-healing performance in sunlight. Mater. Chem. Front. **3**, 1341-1348 (2019). https://doi.org/10.1039/C8QM00607E

[S4] D. Weng, F. Xu, X. Li, Y. Li, J. Sun, Bioinspired photothermal conversion coatings with self-healing superhydrophobicity for efficient solar steam generation. J. Mater. Chem. A **6**, 24441-24451 (2018). https://doi.org/10.1039/C8TA08706G

[S5] X. Li, Y. Li, T. Guan, F. Xu, J. Sun, Durable, highly electrically conductive cotton fabrics with healable superamphiphobicity. ACS Appl. Mater. Interfaces **10**, 12042-12050 (2018). https://doi.org/10.1021/acsami.8b01279

[S6] K. Chen, K. Gu, S. Qiang, C. Wang, Environmental stimuli-responsive self-repairing waterbased superhydrophobic coatings. RSC Adv. **7**, 543-550 (2017). https://doi.org/10.1039/C6RA25135H

[S7] K. Chen, S. Zhou, S. Yang, L. Wu, Fabrication of all-water-based self-repairing superhydrophobic coatings based on UV-responsive microcapsules. Adv. Funct. Mater. **25**, 1035-1041 (2015). https://doi.org/10.1002/adfm.201403496

[S8] H. Wang, Y. Xue, J. Ding, L. Feng, X. Wang et al., Durable, Self-healing superhydrophobic and superoleophobic surfaces from fluorinated-decyl polyhedral oligomeric silsesquioxane and hydrolyzed fluorinated alkyl silane. Angew. Chem. Int. Ed. **50**, 11433- 11436 (2011). https://doi.org/10.1002/anie.201105069

[S9] S. Qiang, K. Chen, Y. Yin, C. Wang, Robust UV-cured superhydrophobic cotton fabric surfaces with self-healing ability. Mater. Des. **116**, 395-402 (2017). https://doi.org/10.1016/j.matdes.2016.11.099

[S10] H. Zhou, H. Wang, H. Niu, A. Gestos, T. Lin, Robust, Self-healing superamphiphobic fabrics prepared by two-step coating of fluoro-containing polymer, fluoroalkyl silane, and modified silica nanoparticles. Adv. Funct. Mater. **23**, 1664-1670 (2013). https://doi.org/10.1002/adfm.201202030

[S11] Y. Wang, X. Wei Liu, H. F. Zhang, Z. P. Zhou, Fabrication of self-healing super-hydrophobic surfaces on aluminium alloy substrates. AIP Adv. **5**, 041314 (2015). https://doi.org/10.1063/1.4905741

[S12] H. Zhou, H. Wang, H. Niu, Y. Zhao, Z. Xu et al., A waterborne coating system for preparing robust, self-healing, superamphiphobic surfaces. Adv. Funct. Mater. **27**, 1604261 (2017). https://doi.org/10.1002/adfm.201604261

[S13] D. Li, Z. Guo, Stable and self-healing superhydrophobic MnO_2@fabrics: Applications in self-cleaning, oil/water separation and wear resistance. J. Colloid Interf. Sci. **503**, 124-130 (2017). https://doi.org/10.1016/j.jcis.2017.05.015

[S14] Y. Sun, Z. Guo, A scalable, self-healing and hot liquid repelling superamphiphobic spray coating with remarkable mechanochemical robustness for real-life applications. Nanoscale **11**, 13853-13862 (2019). https://doi.org/10.1039/C9NR02893E

[S15] K. Chen, J. Zhou, X. Che, R. Zhao, Q. Gao, One-step synthesis of core shell cellulose-silica/n-octadecane microcapsules and their application in waterborne self-healing multiple protective fabric coatings. J. Colloid Interface Sci. **566**, 401-410 (2020). https://doi.org/10.1016/j.jcis.2020.01.106

[S16] Q. Zeng, C. Ding, Q. Li, W. Yuan, Y. Peng et al., Rapid fabrication of robust, washable, self-healing superhydrophobic fabrics with non-iridescent structural color by facile spray coating. RSC Adv. **7**, 8443-8452 (2017). https://doi.org/10.1039/C6RA26526J

[S17] Y. Liu, Z. Liu, Y. Liu, H. Hu, Y. Li et al., One-step modification of fabrics with bioinspired polydopamine@octadecylamine nanocapsules for robust and healable self-cleaning performance. Small **11**, 426-431 (2015). https://doi.org/10.1002/smll.201402383

[S18] C.H. Xue, X. Bai, S.T. Jia, Robust, Self-healing superhydrophobic fabrics prepared by one-step coating of PDMS and octadecylamine. Sci. Rep. **6**, 27262 (2016). https://doi.org/10.1038/srep27262

[S19] H. Wang, Z. Liu, X. Zhang, C. Lv, R. Yuan et al., Durable self-healing superhydrophobic coating with biomimic “chloroplast” analogous structure. Adv. Mater. Interfaces **3**, 1600040 (2016). https://doi.org/10.1002/admi.201600040

[S20] Y. Lee, E.A. You, Y.-G. Ha, Rationally Designed, multifunctional self-assembled nanoparticles for covalently networked, flexible and self-healable superhydrophobic composite films. ACS Appl. Mater. Interfaces **10**, 9823-9831 (2018). https://doi.org/10.1021/acsami.7b19045

[S21] Y. Wang, S. Peng, X. Shi, Y. Lan, G. Zeng et al., A fluorine-free method for fabricating multifunctional durable superhydrophobic fabrics. Appl. Surf. Sci. **505**, 144621 (2020). https://doi.org/10.1016/j.apsusc.2019.144621

[S22] Y. Fu, F. Xu, D. Weng, X. Li, Y. Li et al., Superhydrophobic foams with chemical- and mechanical-damage-healing abilities enabled by self-healing polymers. ACS Appl. Mater. Interfaces **11**, 37285-37294 (2019). https://doi.org/10.1021/acsami.9b11858

[S23] Y.P. Liu, H.F. Liu, Y.G. Feng, Z.L. Liu, H.Y. Hu et al., A nanotubular coating with both high transparency and healable superhydrophobic self-cleaning properties. RSC Adv. **6**, 21362-21366 (2016). https://doi.org/10.1039/C5RA26977F

[S24] M. Wu, B. Ma, T. Pan, S. Chen, J. Sun, Silver-nanoparticle-colored cotton fabrics with tunable colors and durable antibacterial and self-healing superhydrophobic properties. Adv. Funct. Mater. **26**, 569-576 (2016). https://doi.org/10.1002/adfm.201504197

[S25] S. Chen, X. Li, Y. Li, J. Sun, Intumescent flame-retardant and self-healing superhydrophobic Coatings on Cotton Fabric. ACS Nano **9**, 4070-4076 (2015). https://doi.org/10.1021/acsnano.5b00121

[S26] D. Zhu, X. Lu, Q. Lu, Electrically conductive PEDOT coating with self-healing superhydrophobicity. Langmuir **30**, 4671-4677 (2014). https://doi.org/10.1021/la500603c

[S27] M. Wu, N. An, Y. Li, J. Sun, Layer-by-layer assembly of fluorine-free polyelectrolyte–surfactant complexes for the fabrication of self-healing superhydrophobic films. Langmuir **32**, 12361-12369 (2016). https://doi.org/10.1021/acs.langmuir.6b02607

[S28] Y. Li, S. Chen, M. Wu, J. Sun, All spraying processes for the fabrication of robust, self-healing, superhydrophobic coatings. Adv. Mater. **26**, 3344-3348 (2014). https://doi.org/10.1002/adma.201306136

[S29] Y. Li, Y. Zhao, X. Lu, Y. Zhu, L. Jiang, Self-healing superhydrophobic polyvinylidene fluoride/Fe_3O_4@polypyrrole fiber with core–sheath structures for superior microwave absorption. Nano Res. **9**, 2034-2045 (2016). https://doi.org/10.1007/s12274-016-1094-x

[S30] S. Liu, N. Masurkar, S. Varma, I. Avrutsky, L.M. Reddy Arava, Experimental studies and numerical simulation of polypyrrole trilayer actuators. ACS Omega **4**, 6436-6442 (2019). https://doi.org/10.1021/acsomega.9b00032

[S31] A. Majumdar, S. Mukhopadhyay, R. Yadav, Thermal properties of knitted fabrics made from cotton and regenerated bamboo cellulosic fibres. Int. J. Therm. Sci. **49**, 2042-2048 (2010). https://doi.org/10.1016/j.ijthermalsci.2010.05.017

[S32] Specific Heat of Solids. https://www.engineeringtoolbox.com/specific-heat-solids-d_154.html (accessed June 2021).

[S33] F.S.C. Mustata, A. Mustata, Dielectric behaviour of some woven fabrics on the basis of natural cellulosic fibers. Adv. Mater. Sci. Eng. **2014**, 216548 (2014). https://doi.org/10.1155/2014/216548

[S34] Properties of cotton. https://barnhardtcotton.net/technology/cotton-properties/(accessed June 2021).
